# Nudging parents and teachers to improve learning and reduce child labor in Cote d’Ivoire

**DOI:** 10.1038/s41539-023-00180-z

**Published:** 2023-09-13

**Authors:** Sharon Wolf, Guilherme Lichand

**Affiliations:** 1 University of Pennsylvania, Philadelphia, PA USA; 2 Stanford Graduate School of Education, Palo Alto, CA, USA

**Keywords:** Education, Economics

## Abstract

Whether SMS-based nudge interventions can increase parent engagement and improve child learning outcomes across diverse contexts such as rural West Africa is unknown. We conducted a school-randomized trial to test the impacts of an audio or text-message intervention (two messages per week for one school year) to parents and teachers of second and fourth grade students (*N* = 100 schools, 2246 students) in Cote d’Ivoire. Schools were randomly assigned to have messages sent to (i) parents only, (ii) teachers only, (iii) parents and teachers together, or (iv) control. There were statistically non-significant impacts of the parents-only treatment on learning, although with typical effect sizes (*d* = 0.08, *p* = 0.158), and marginally statistically significant increases in child labor (*d* = 0.11, *p* < 0.10). We find no impacts of the other treatment conditions. Subgroup analyses based on pre-registered subgroups show significantly larger improvements in learning for children with below-median baseline learning levels for the parents-only arm and negative impacts on learning for girls for the teachers-only arm, suggesting different conclusions regarding impacts on equity for vulnerable children.

## Introduction

Over the next century, the largest increase in the world population will take place in Africa^1^. This fast-rising population brings significant opportunities, and improving human capital through education will be critical. Countries across sub-Saharan Africa have made remarkable progress in increasing enrollment rates in primary school over the past two decades, yet learning outcomes remain very low^2^. Worldwide, it is estimated that 160 million children engage in child labor, accounting for almost 10% of the overall child population, with around half engaged in hazardous work^3^. The vast majority of child labor in developing countries is rural and agricultural, and in this context, children tend to balance both labor and schooling (International Labour Organization, 2021). These children are less likely to attend school regularly and become literate than their peers^4^.

Parents provide a foundation for children’s academic success through beliefs and expectations they hold for their children, as well as direct engagement in children’s learning at home and in school and their time use. Changing educational investments in rural agricultural settings where poverty levels are high may require addressing the barriers parents face related to their own limited schooling experience and economic demands. Evidence from behavioral economics indicates that “nudges” that target informational barriers and increase the salience of education for parents hold promise^5^. At the same time, if parents encourage school engagement but teacher motivation and educational quality are low due to poor training, increased workloads, and lack of professional support, increased engagement may not result in improved learning.

In this study, we report results from an impact evaluation examining whether a program that delivered nudges via text messages (SMS) and audio messages to parents and teachers impacted child learning and labor outcomes in rural Cote d’Ivoire (see Supplementary Materials for more details on the association between education and child labor). Messages to parents aimed to increase the salience of and investment in education, while messages to teachers aimed to increase motivation and improve teaching practice. Overall, we found no statistically significant impacts on improved learning outcomes, but significant variation by subgroups. Messages to parents alone produced a more positive pattern of impacts than messages to teachers and both parents and teachers together. The results have implications for how low-cost, scalable strategies that build on theory from behavioral economics may support children’s education in marginalized communities in sub-Saharan Africa.

There are well-documented gaps between how advantaged and disadvantaged parents invest in children’s education across high-^6^ and low- and middle-income countries^7,8^. Recent insights from behavioral science point to some of the barriers low-income and low-educated parents face that contribute to this gap, despite having relatively equal aspirations for their children’s education^9^. First, the payoff in educational investments does not materialize for many years into the future, requiring parents to make temporal tradeoffs between present-day priorities. Second, parenting requires an understanding of children’s thoughts, feelings and preferences, and such parental attributions influence parent–child interactions^10^. Third, parenting decisions are often automatic rather than deliberate, which reduces cognitive load but also leads to rapid and repeated responses in many situations. Fourth, parenting decisions are experienced as identity-relevant, and parents' own experiences with schooling and their own literacy may play a key role in the investments they make in their children.

Most parenting interventions try to change parenting behaviors through in-person education sessions that are often resource- and time-intensive, and rarely directly target these behavioral barriers. Programs that aim to breakdown parenting behaviors into smaller steps and provide parents with small bits of timely and actionable information over time hold promise. This approach can reduce barriers to behavior change and improve parental engagement. Similarly, delivering this information via text messaging—a scalable and relatively cheap platform—has proven effective in high- and middle-income country contexts^11–14^. While evidence from low-income countries is scarce, a recent evaluation in Botswana found that receiving regular SMS messages and phone calls together (but not SMS messages alone) to guide parents on math instruction implemented when schools were closed did lead to small and meaningful improvements in children’s foundational math skills^15^. In Zambia, SMS messages focused on primary schoolchildren’s literacy skills coupled with monthly meetings to encourage program engagement had positive impacts on children’s reading skills of around 0.2 standard deviations^16^. In Brazil, SMS nudges to engage parents of secondary school students in their children’s school life increased standardized test scores by 0.1 standard deviations and decreased grade repetition rates by one-third^17^. Differences between the Brazilian and the Ivorian contexts—from parents’ literacy rates to teacher absenteeism—make it an open question whether a similar intervention would work in the context of this study. Importantly, the two studies in SSA included additional support to parents beyond the SMS messages.

Mobile messaging to teachers has also been proposed as a cost-effective way to support teachers both to provide pedagogical information and increase motivation^18^, though a recent report concluded that more evidence is needed on how programs implemented as complements to schooling^19^. The underlying presumption is that teaching quality is low due to a lack of information about effective pedagogy (which can be delivered in small-sized bits through messages) and that the lack of support and professional development is de-motivating to teachers. If crafted effectively, SMS messages may address both issues. For example, in Niger, adding weekly phone calls to teachers, village chiefs, and students increased the effectiveness of a teacher education program on student learning outcomes by 0.15 standard deviations, suggesting that the calls increased teacher motivation^20^. A synthesis review of the evidence from developing countries concluded that simple but effective strategies to enhance teachers’ practices and motivation have been demonstrated using SMS^21^. Yet most evaluations do not disentangle the impacts of the messages with other components of teacher professional development, as they are often incorporated as a complement to in-person professional development activities.

We focus on children in cocoa-growing areas of Cote d’Ivoire, a West African country with a population of 25.7 million people with a life expectancy of 57 years^22^. The country ranks 170 of 189 countries on the Human Development Index (a composite index of life expectancy, education, and per capita income) and is the largest producer of cocoa in the world^23^. In rural cocoa-producing communities, poverty is rampant^24^, with many households living on $1–2 a day^25^.

Ivorian cocoa production is mostly maintained by small family farmers who mainly use family labor^26^. Recent estimates are that 1.56 million children between the ages of 5 and 17 are working in cocoa production in Ghana and Cote d’Ivoire, with increases over the past decade despite large-scale efforts to reduce child labor^27^. Based on representative survey interviews conducted with 1409 household head surveys, 2734 child surveys across 15 regions, 80% of children in agricultural households in Cote d’Ivoire are enrolled in school, with 11% of children working in cocoa production not attending school, and additional 6% of children working in cocoa production reporting that work interfered with schooling^27^.

Very little research has been conducted on barriers to parental investment in children’s education in the rural Ivorian context. But parental education is generally low and poverty is widespread^28^, and parents likely make temporal tradeoffs between present-day priorities that lead to an immediate reduction in hardship and stress. Further, high enrollment rates suggest that parents value schooling, but there is little data on family-level processes that support child schooling outcomes. Research with poor parents in Ghana, a country neighboring Cote d’Ivoire, indicates that parents deeply value their child’s education but see their role as one that focuses on providing necessities and not engaging directly with their child’s education and learning^29^. These findings point to the role of identity-relevance in the decisions parents make, though few studies have examined mediating mechanisms through which nudge-based message programs work. Examining how such interventions shape motivation to engage with children, as well as actual involvement at home and school, could help shed light on some of these pathways.

While the Ivorian government has committed to expanding educational access through universal basic education, teaching quality, and learning outcomes in Cote d’Ivoire are very low, particularly in poor rural cocoa-growing regions. The most recent PASEC data, which focuses on Francophone countries in West Africa, rates Cote d’Ivoire among the bottom 30 countries globally^2^, with large inequalities between urban and rural regions^30^. This is partly due to poor teacher motivation and performance and in rural areas very large class sizes and little ongoing professional development and training for teachers in hard-to-reach schools^31^. Even among children attending primary school, the average fifth grader can only read a few words^4^.

Children’s sex is a key determinant for child labor and schooling, with boys more likely to engage in labor^32^. Boys are also more likely to balance both work and school, whereas girls are more likely to specialize in one or the other^33^. Further, parent engagement may vary by child sex due to greater opportunity costs of schooling for girls (e.g., larger involvement of girls in household or care work), lower perceived returns to girls’ education, and widespread gender bias in social norms and aspirations^34^. Current school enrollment rates in Cote d’Ivoire are 86.0%, with disparities between males and females at 89.8% and 82.1%, respectively. The sex gap is more pronounced in secondary school, with 44.5% and 33.4% net enrollment for males and females, respectively^35^.

Research shows that the poorest parents often have less accurate information about their children’s academic abilities; when given more accurate information about their children, parents increase the school enrollment of their higher-performing children, decrease the enrollment of lower-performing children, and choose educational inputs that are more closely matched to their children’s academic level^36^. At the same time, evidence from research in early childhood education shows that the benefits of quality education may be greatest for children with low initial skills^37^. If a program aiming to increase parental engagement generally may have different effects based on children’s ability levels, and in which direction, is not known.

This study evaluated the effectiveness of a message-based nudge intervention (audio and text)—Eduq+—to parents and teachers in second and fourth-grade primary classrooms in rural Cote d’Ivoire to improve children’s learning outcomes and reduce child labor. We examined three primary research questions related to the recipient of the intervention (1–3) and a secondary research question related to heterogeneous treatment effects (4):

1. What are the impacts of the Eduq+ program delivered to parents on children’s literacy and numeracy outcomes and engagement in child labor?
2. What are the impacts of the Eduq+ program delivered to teachers on children’s literacy and numeracy outcomes and engagement in child labor?
3. What are the impacts of the Eduq+ program delivered to both parents and teachers on children’s literacy and numeracy outcomes and engagement in child labor?
4. Do impacts vary by child sex, baseline ability, and parent education?

## Results

### Impacts on learning outcomes

Our primary outcome is a standardized summary measure of the literacy and numeracy skills of children (Table 1). The first column in Table 1 presents the impact estimates for this summary measure, and the second and third columns show the impacts on the individual components of literacy and numeracy, respectively. All outcomes can be interpreted as standard deviation units. There were meaningful but statistically insignificant impacts of the parent treatment on a learning summary measure (*d* = 0.081, *p* = 0.158), and similarly sized but statistically insignificant impacts on each of the two learning domains (for literacy and numeracy, *p* = 0.236 and 0.108, respectively). Impacts of the teacher treatment and of the parent and teacher combined treatment on all three outcomes were also statistically insignificant and negative (*d* = −0.019 to −0.073). For reference, learning in the control group was 0.33 SD over the course of the year, suggesting that these impacts were meaningful and equivalent to approximately one-quarter of a school year—the typical effect sizes of educational nudges across high- and middle-income countries^5^.

**Table 1:** Impacts on learning outcomes and child labor.

	Learning summary measure	Literacy	Numeracy	Child-reported child labor
	*b*/(SE)/*p*			
Parents	0.081	0.082	0.080	0.113*
	(0.057)	(0.074)	(0.049)	(0.066)
	0.158	0.268	0.108	0.091
Teachers	−0.037	−0.073	0.000	0.012
	(0.048)	(0.061)	(0.047)	(0.069)
	0.449	0.234	0.996	0.863
Both	−0.019	−0.049	0.011	−0.026
	(0.045)	(0.055)	(0.045)	(0.067)
	0.673	0.382	0.811	0.700
Observations	2246	2246	2246	2246
R-squared	0.572	0.533	0.476	0.141
Control mean endline	0.317	0.340	0.295	0.030
Parents = Both [*p*-value]	0.051	0.045	0.142	0.072
Teachers = Both [*p*-value]	0.663	0.619	0.810	0.633

Parents = 1 in schools where parents alone receive message; Teachers = 1 in schools where teachers alone receive messages; Both = 1 in schools where both parents and teachers receive messages. In column (1), the outcome learning summary measure is the average of numeracy and literacy standardized test scores. In column (2), literacy is a standardized summary measure constructed from individual components consisting of French exercises completed by students. In column (3), numeracy is a standardized summary measure constructed from individual components consisting of mathematics exercises completed by students. In column (4), an index of four activities as reported by child as to whether they engaged in the following activities for one or more hours: domestic work, such as buying food or cooking, cleaning the house, do the laundry, take care of children or other sick or old relatives. For each outcome, we follow Kling et al. (2007)^55^ by standardizing each component within grade (normalizing by their mean and standard deviation among the control group) and then averaging across them to construct the summary variable. In all columns, observations are weighted by the inverse of the predicted probability of being tracked at endline, computed using baseline students’ characteristics in the control group. Baseline controls include grade level; child sex; standardized baseline grades (numeracy and literacy); standardized parental engagement; standardized student effort; standardized child labor; standardized socio-emotional skills; standardized working memory; standardized visual attention; standardized impulsivity; standardized self-esteem; standardized mindset. Standard errors clustered at the school level in parentheses. *p < 0.1.

### Impacts on child labor

The fourth column of Table 1 presents impacts on child labor as reported by children. Results show that in the parent-only treatment, children report a marginally statistically significant increase in child labor (*d* = 0.113, *p* = 0.091). There were no impacts of the other two treatment arms on child-reported child labor, and point estimates were close to zero.

We re-ran our models changing the reference group to compare whether the magnitude of effects differed across the three treatment arms. Results showed differences between the parents-only and combined treatment arms, with larger effects for the parent-only arm. Specifically, differences in the impact on child-reported labor were statistically significant (*d* = 0.138, SE = 0.076, *p* = 0.072).

### Impact heterogeneity by child sex, baseline skills, and parent education

**Fig. 1:**
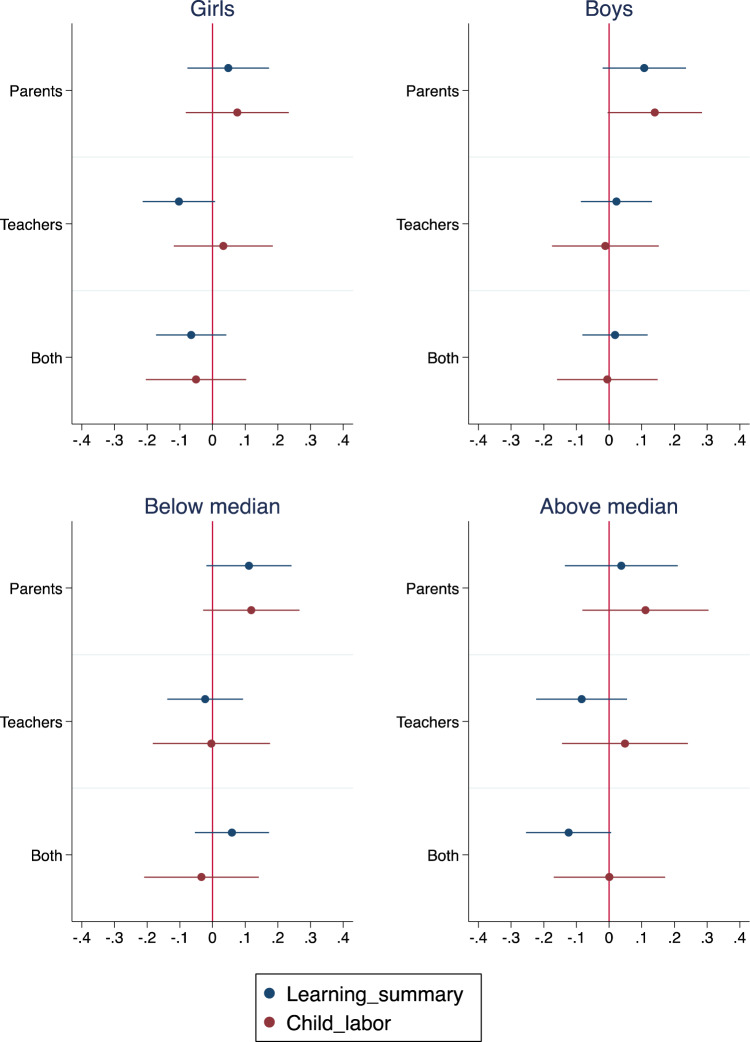
Coefficient plots for impact estimates on primary outcomes by child sex and baseline test score. Circles represent impact estimate coefficients for the respective treatment arm and subgroup; lines represent 95% confidence intervals.

Tables 2–4 present impact heterogeneity tests, with coefficient plots by child sex and baseline skills shown in Fig. 1. There was some evidence that the treatment arms that targeted teachers yielded better outcomes for boys and even had some negative effects on girls’ learning. Specifically, there was a statistically significant interaction effect for the teacher’s only condition with child sex in predicting summary learning scores (*ß* = −0.13, *p* < 0.05), and for the combined arm in predicting literacy scores (*ß* = 0.12, *p* < 0.10; see Table 2).

**Table 2:** Impact variation by child sex.

	Learning summary measure	Literacy	Numeracy	Child labor
	*b*/(SE)/*p*			
Parents	0.108*	0.095	0.121**	0.147**
	(0.063)	(0.078)	(0.059)	(0.073)
	0.088	0.225	0.043	0.046
Parents × Girl	−0.061	−0.034	−0.088	−0.067
	(0.053)	(0.065)	(0.059)	(0.077)
	0.250	0.598	0.142	0.387
Teachers	0.027	−0.026	0.081	−0.009
	(0.055)	(0.073)	(0.055)	(0.081)
	0.622	0.723	0.144	0.912
Teachers × Girl	−0.132**	−0.099	−0.166***	0.039
	(0.055)	(0.079)	(0.055)	(0.080)
	0.017	0.214	0.003	0.623
Both	0.019	0.009	0.029	0.001
	(0.050)	(0.060)	(0.051)	(0.078)
	0.706	0.883	0.580	0.992
Both × Girl	−0.081	−0.121*	−0.042	−0.054
	(0.054)	(0.063)	(0.064)	(0.079)
	0.139	0.059	0.513	0.495
Girl	0.059	0.089*	0.029	−0.063
	(0.040)	(0.046)	(0.047)	(0.057)
	0.138	0.054	0.536	0.271
Observations	2246	2246	2246	2246
Control mean endline girls	0.358	0.409	0.306	−0.034
Control mean endline boys	0.282	0.279	0.285	0.085

Parents = 1 in schools where parents alone receive message; Teachers = 1 in schools where teachers alone receive messages; Both = 1 in schools where both parents and teachers receive messages. In column (1), the outcome learning summary measure is the average of numeracy and literacy standardized test scores. In column (2), literacy is a standardized summary measure constructed from individual components consisting of French exercises completed by students. In column (3), numeracy is a standardized summary measure constructed from individual components consisting of mathematics exercises completed by students. In column (4), an index of four activities as reported by child as to whether they engaged in the following activities for one or more hours: domestic work, such as buying food or cooking, cleaning the house, do the laundry, take care of children or other sick or old relatives. For each outcome, we follow Kling et al. (2007)^55^ by standardizing each component within grade (normalizing by their mean and standard deviation among the control group) and then averaging across them to construct the summary variable. In all columns, observations are weighted by the inverse of the predicted probability of being tracked at endline, computed using baseline students’ characteristics in the control group. Baseline controls include grade level; child sex; standardized baseline grades (numeracy and literacy); standardized parental engagement; standardized student effort; standardized child labor; standardized socio-emotional skills; standardized working memory; standardized visual attention; standardized impulsivity; standardized self-esteem; standardized mindset. Standard errors clustered at the school level in parentheses.

***p < 0.01, **p < 0.05, *p < 0.1.

**Table 3:** Impact variation by baseline test scores.

	Learning summary measure	Literacy	Numeracy	Child labor
	*b*/(SE)/*p*			
Parents	0.115*	0.141*	0.088	0.121*
	(0.066)	(0.074)	(0.068)	(0.072)
	0.086	0.059	0.202	0.094
Parents × Above median	−0.081	−0.142	−0.021	−0.017
	(0.103)	(0.135)	(0.086)	(0.109)
	0.431	0.297	0.807	0.877
Teachers	−0.018	−0.083	0.046	−0.002
	(0.061)	(0.055)	(0.078)	(0.089)
	0.764	0.140	0.558	0.979
Teachers × Above median	−0.064	−0.024	−0.104	0.042
	(0.091)	(0.111)	(0.092)	(0.130)
	0.486	0.827	0.264	0.748
Both	0.059	0.072	0.046	−0.025
	(0.057)	(0.060)	(0.071)	(0.087)
	0.304	0.234	0.517	0.775
Both × Above median	−0.173**	−0.262**	−0.084	0.019
	(0.087)	(0.104)	(0.091)	(0.113)
	0.049	0.014	0.355	0.868
Above median	0.151**	0.215***	0.087	−0.078
	(0.064)	(0.081)	(0.059)	(0.064)
	0.019	0.009	0.140	0.222
Observations	2246	2246	2246	2246
Control mean, below median	0.037	−0.013	0.086	0.101
Control mean, above median	0.699	0.820	0.578	−0.067

Parents = 1 in schools where parents alone receive message; Teachers = 1 in schools where teachers alone receive messages; Both = 1 in schools where both parents and teachers receive messages. In column (1), the outcome learning summary measure is the average of numeracy and literacy standardized test scores. In column (2), literacy is a standardized summary measure constructed from individual components consisting in French exercises completed by students. In column (3), numeracy is a standardized summary measure constructed from individual components consisting of mathematics exercises completed by students. In column (4), an index of four activities as reported by child as to whether they engaged in the following activities for one or more hours: domestic work, such as buying food or cooking, cleaning the house, do the laundry, take care of children or other sick or old relatives. For each outcome, we follow Kling et al. (2007)^55^ by standardizing each component within grade (normalizing by their mean and standard deviation among the control group) and then averaging across them to construct the summary variable. In all columns, observations are weighted by the inverse of the predicted probability of being tracked at endline, computed using baseline students’ characteristics in the control group. Baseline controls include grade level; child sex; standardized baseline grades (numeracy and literacy); standardized parental engagement; standardized student effort; standardized child labor; standardized socio-emotional skills; standardized working memory; standardized visual attention; standardized impulsivity; standardized self-esteem; standardized mindset. Standard errors clustered at the school level in parentheses.

*p < 0.1, **p < 0.05, ***p < 0.01.

**Table 4:** Impact variation by parent education.

	Learning summary measure	Literacy	Numeracy	Child-reported labor
	*b*/(SE)/*p*			
Parents	0.102	0.085	0.119*	0.142**
	(0.074)	(0.095)	(0.063)	(0.067)
	0.171	0.378	0.064	0.038
Parents × Unschooled parents	0.011	0.059	−0.037	−0.063
	(0.067)	(0.097)	(0.056)	(0.088)
	0.872	0.545	0.504	0.480
Teachers	0.003	−0.044	0.050	0.036
	(0.064)	(0.086)	(0.055)	(0.074)
	0.965	0.965	0.965	0.965
Teachers × Unschooled parents	−0.076	−0.022	−0.131**	−0.068
	(0.065)	(0.092)	(0.056)	(0.075)
	0.245	0.814	0.021	0.367
Both	−0.025	−0.054	0.005	−0.011
	(0.070)	(0.099)	(0.055)	(0.072)
	0.724	0.586	0.929	0.881
Both × Unschooled parents	0.079	0.101	0.057	−0.020
	(0.088)	(0.126)	(0.068)	(0.083)
	0.371	0.423	0.402	0.807
Unschooled parents	−0.035	−0.091	0.021	0.151**
	(0.055)	(0.080)	(0.042)	(0.058)
	0.530	0.254	0.613	0.010
Observations	1962	1962	1962	1962
Control mean, no schooling	0.191	0.145	0.236	0.165
Control mean, any schooling	0.365	0.438	0.292	−0.046

Parents = 1 in schools where parents alone receive message; Teachers = 1 in schools where teachers alone receive messages; Both = 1 in schools where both parents and teachers receive messages. In column (1), the outcome learning summary measure is the average of numeracy and literacy standardized test scores. In column (2), literacy is a standardized summary measure constructed from individual components consisting in French exercises completed by students. In column (3), numeracy is a standardized summary measure constructed from individual components consisting of mathematics exercises completed by students. In column (4), an index of four activities as reported by child as to whether they engaged in the following activities for one or more hours: domestic work, such as buying food or cooking, cleaning the house, do the laundry, take care of children or other sick or old relatives. For each outcome, we follow Kling et al. (2007)^55^ by standardizing each component within grade (normalizing by their mean and standard deviation among the control group) and then averaging across them to construct the summary variable. In all columns, observations are weighted by the inverse of the predicted probability of being tracked at endline, computed using baseline students’ characteristics in the control group. Baseline controls include grade level; child sex; standardized baseline grades (numeracy and literacy); standardized parental engagement; standardized student effort; standardized child labor; standardized socio-emotional skills; standardized working memory; standardized visual attention; standardized impulsivity; standardized self-esteem; standardized mindset. Standard errors clustered at the school level in parentheses.

*p < 0.1, **p < 0.05.

Table 3 presents the results of impact heterogeneity by child baseline skills (below and above the median at baseline. The pattern of results suggests that impacts were larger and positive for children who performed below the median at baseline (see Table 3 and Fig. 1).

Finally, Table 4 presents results by parent education. Overall, there was not a clear pattern of impact heterogeneity for these subgroups.

### Potential mechanisms

In an exploratory analysis, we examined three potential mechanisms through which the intervention may have shaped parenting as pathways to treatment effects on children: motivation, social-emotional engagement, and school engagement. There were no statistically significant treatment impacts (see Table 5).

**Table 5:** Impacts on potential parenting mechanisms.

	Autonomous motivation	Social-emotional engagement	School engagement
	*b*/SE/*p*		
Parents	0.127	0.122	−0.015
	(0.094)	(0.094)	(0.095)
	0.272	0.665	0.879
Teachers	0.115	0.005	0.005
	(0.093)	(0.091)	(0.023)
	0.286	0.956	0.953
Both	−0.100	−0.135	0.023
	(0.123)	(0.106)	(0.097)
	0.616	0.691	0.815
Observations	2,179	2,179	2,179
R-squared	0.016	0.012	0.006
Control mean endline	0.000	0.000	0.000
Parents = Both [*p*-value]	0.079	0.032	0.712
Teachers = Both [*p*-value]	0.096	0.234	0.849

Parents = 1 in schools where parents alone receive message; Teachers = 1 in schools where teachers alone receive messages; Both = 1 in schools where both parents and teachers receive messages. In column (1), motivation is a standardized factor score based on nine individual items and standardized relative to the control group. In columns (2) and (3), engagement is computed through summing up responses on nine items for each scale, respectively, about the frequency in which parents engage in a set of activities with children. In all columns, observations are weighted by the inverse of the predicted probability of being tracked at endline, computed using baseline students’ characteristics in the control group. Baseline controls include grade level; child sex; standardized baseline grades (numeracy and literacy); standardized parental engagement; standardized student effort; standardized child labor; standardized socio-emotional skills; standardized working memory; standardized visual attention; standardized impulsivity; standardized self-esteem; standardized mindset. Standard errors clustered at the school level in parentheses.

## Discussion

We report on the impacts of a low-cost message-based intervention that aimed to change parent and teacher investments in education in rural cocoa-farming communities in Cote d’Ivoire. Nudge-based interventions delivered solely via text messages for parents have recently been shown to change parental investments and child outcomes in the United States^12^, Brazil^17^, and Costa Rica^15^. But the effectiveness of similar programs in a rural West African context, where parental literacy and schooling levels are low, their utility for teachers, and their application to impacting child labor, has not been tested previously. Our results show if implemented without additional support in a rural West African context, such programs may have limited impacts, and call attention to the need to examine impact variation with a focus on vulnerable groups of children. Further, targeting parents with such programs may yield more positive effects than targeting teachers or parents and teachers together.

The content of the messages in the program we tested aimed to increase parents’ engagement in their child’s education and social-emotional development. We found evidence of small increases in children’s academic skills of children who performed at the bottom half of their class at baseline, indicating that despite parents’ low schooling and literacy levels, the intervention likely led to some meaningful changes in the home for this group of children. These average effect sizes are somewhat smaller and estimated with more uncertainty than what has been found in other studies that have tested similar programs on children’s literacy and numeracy outcomes (*d* = 0.10–0.25^5^), but learning over the course of one school year is much lower in Cote d’Ivoire (i.e., the control group learned about one-third of a standard deviation from baseline to endline). We cannot conclude whether the smaller effect sizes in our study are due to parent characteristics in our sample, the study context, or the intervention itself, though the intervention tested was similar to the program in Brazil which found effects of a similar size^17^. A recent study implemented during school closures in Botswana found that a more intensive phone-based program comprising messages and phone calls to parents was effective in improving children’s math skills (with effect sizes around 0.12^15^).

Interestingly, there were no differences between messaging parents in simple French text messages compared to audio messages in the local language. This is surprising given that the adult literacy rate in Cote d’Ivoire is 47.2%^30^, and likely lower in rural communities. More implementation data is needed to understand how parents are engaged with the messages. For example, did parents ask a literate household member or neighbor for help reading the message? Did they go to the school to ask the child’s teacher for help? Given how few studies have implemented similar programs in rural African communities, these are important questions for future research to examine.

One innovation of our paper is that we tested select mechanisms of change at the parent-level. We found no main effects on motivation for parenting, but subgroup analyses (not shown) showed that impacts on engagement were positive for girls and children with baseline learning scores below the median. Further, impacts on autonomous motivation were positive and larger for lower-performing children. These findings warrant further investigations to understand why messages shifted parents’ beliefs and behaviors differently for some subgroups but not others, and why these did not always translate into a similar pattern of benefits for children’s learning outcomes. The program may have addressed some barriers to educational investments, perhaps such as attributions and identity-relevance, but not others such as temporal tradeoffs and automatic decision-making^9^, suggesting that parents may need additional support to change the quantity and nature of their time investments with all children. Notably, our exploratory analysis of mediators was limited, and future research is needed to understand how parents interact with and interpret messages in nudge-based programs. An explicit focus on parental mechanisms—including how their beliefs, aspirations, and behaviors do or do not change—is necessary to optimize such programs across diverse populations.

Another contribution of this study was the factorial design testing the added benefits of sending similar nudge-based messages to teachers to increase motivation and effectiveness and to parents and teachers together. Teachers in rural Ivorian schools receive little professional support and development, and poor educational quality and poor teacher motivation are major barriers to children’s learning in sub-Saharan Africa^31^. We did not find a clear pattern of impacts on teachers, suggesting that more intensive efforts are likely needed to improve teaching quality. A review of the literature on teacher professional development from high-income countries concluded that effective programs include a content-specific focus, support active learning and collaboration among teachers, and provide models of effective practice and coaching and support with feedback and reflection^38^. Thus, SMS messages may be a good complement to programs focused more on content and in-person support. In another study, we show that in schools where both teachers and parents were nudged, teacher attendance *decreased*, particularly for teachers who had high levels of motivation at baseline, and this was a function of increased parental monitoring of teachers^39^. This suggests that engaging parents and teachers at too high of an intensity can backfire and ultimately lead to teachers feeling more de-motivated. Similar findings have been shown in Mexico^40^.

We also examined impact heterogeneity by children’s baseline learning skills, parent education, and child sex. First, the impacts of the parent-only arm on learning outcomes were larger for children with below-median baseline learning scores, suggesting that the program targeting parents had a larger effect on lower-performing children. We were not able to collect child-level data on grade repetition or dropout rates, but in a companion paper we found that at the aggregate school-level, treatment impacts of the parent-only and teachers-only treatment arms reduced dropout rates, and these impacts were largest for older children^39^. Second, we found no differences by parent education level (i.e., some formal schooling versus none), contrary to another study in Ghana which found larger effects for parents with formal schooling^41^. Third, we found that the overall pattern of impacts was more positive for boys than girls. The teacher-only program had negative effects on girls’ learning, a concerning outcome that should be examined further in future studies.

Finally, a contribution of this study was to examine the potential of nudge-based messages to parents to reduce child labor, an issue that has received growing international attention in recent years. For children under 12 years of age, any form of work/employment is considered child labor^42^. Using child reports of engagement in four different types of labor activities in the previous month, we found marginally statistically significant *increases* in child labor participation, contrary to the goals of the intervention. In cocoa-farming communities, close to 40% of children engage in farm labor; a large majority (80%) of those children are also enrolled in school^27^, and our results reflect a similar pattern. The Eduq+ messages that targeted child labor specifically focused on cocoa farming. Interestingly, the increases we observed were in domestic and construction work and *not* cocoa farming activities (see Supplementary Materials). Parents may not view this type of work as labor, but rather as a form of children’s socialization and family responsibilities^43,44^. It is plausible that the messages from the intervention increased salience for parents of their child’s capabilities^17^, and thus increased attention to children’s socialization more generally, including demands on children in terms of both their schooling and domestic expectations. Much more research is needed to understand how engagement in labor activities intersects with children’s schooling outcomes given how widespread both are in this context.

Accurately measuring child labor is not straightforward. One issue is social desirability bias, where respondents may assume it is less desirable to report engaging in labor given child labor laws, thus under-report. Large-scale surveys conducted in West Africa on child labor rely on child reports^27^, and another study of ours suggests that child-reports are more accurate than parent- and teacher-reports, as verified by satellite imagery documenting child labor within communities^45^. We rely on child-reports for this reason, though our measures were simplistic, asking children to report on engagement in four activities in the past month. A second issue is measuring the frequency and duration of children’s engagement in such work. There are likely to be different consequences for children who work on their family farm for a few hours a week compared to those who work on the farm for several hours each day. In addition, distinguishing between engagement in hazardous and non-hazardous work is important. The consequences of hazardous work—including chemical exposure, injuries, and illnesses including fractures and lacerations—can be more serious and consequential for school engagement^26^. But obtaining accurate estimates of the frequency, as well as the types of tasks children engage in, is challenging and requires lengthy surveys dedicated solely to measuring engagement in labor. Additional longitudinal research is needed to understand how children are balancing labor activities and school, and how different interventions affect this balance.

This study has several limitations that should be considered when interpreting the results. The first and most important is that as a school-randomized trial with four treatment arms, the study was underpowered to detect the size of effects generally found in the literature on nudge-based SMS interventions. Concretely, we were underpowered to detect the small impacts detected on learning outcomes observed, which are meaningful in terms of translation into over 2 months of learning. Second, the sample is limited to two regions in Cote d’Ivoire and is not representative of all cocoa-growing regions in the country. Further, the schools selected for the study were not randomly sampled but rather selected by the regional education offices. Thus, generalizability is limited. Second, our data on mechanisms of change was limited, and it would be critical for future research to examine a larger set of potential pathways to further understand how such interventions can change parent–child, teacher–child, and parent–teacher relationships to understand how to better target programs to achieve larger and more robust effects. Our study cannot speak to these important issues and thus we are left speculating about the pathways of treatment effects. Finally, we cannot address the importance of the content in generating change. Specifically, the sequence of the program included a motivating fact, a suggested activity, an interactive message, and a growth message, and we do not know whether all four features of the texting program have a larger effect than the sum of the components^17^ study how the frequency, time of delivery and interactivity of messaging matters for impacts in Brazil). Our inability to understand the key “ingredients” of change limits the conclusions we can draw from the study.

Nonetheless, this study provides important insights into message-based nudge interventions for parents and teachers in rural West Africa. The results suggest that there is potential for such programs to play a role in broader efforts to improve educational quality and learning in these contexts, but caution that attention must be paid to heterogenous effects for different groups of vulnerable children.

Assessing impacts relative to program costs is critical. The costs involved to develop the program, enroll parents, oversee the project, and other operational expenses came out to 796 USD per school or 6.68 USD per student. If the program were to be implemented again, the only costs would be enrollment of schools and parents (which can be done over the phone quite cheaply or through the Ministry of Education for free), and the costs of sending two SMS messages per week (anywhere from 0.03 to 0.065 cents per message in 2021 in Cote d’Ivoire). Alternatively, the Ministry of Education could coordinate with the carriers to send SMS for free or use UNICEF’s RapidPro infrastructure to send SMS for no cost.

There is promise in these programs, though our results suggest that without additional support to parents and teachers, their effects are likely to be limited. Importantly, the program targeting parents alone yielded more positive benefits for children with lower learning levels at baseline, suggesting that these programs may address some parental barriers to educational engagement. In contrast, the program targeting teachers had few positive benefits for children and even some negative effects on learning for girls, suggesting that teachers may need different supports to improve educational practice, equity, and ultimately child learning outcomes. To increase effects on learning, demand-side programs such as this one likely need to be complemented by supply-side programs (i.e., teacher professional development) to translate into meaningful gains for children’s learning. Finally, our results that the program to parents increased child labor, and domestic labor specifically, indicate that parents’ views on child labor will need to be considered in future iterations of such programs to design them more optimally. Further research is needed to understand how to optimize nudge-based programs to support children’s learning in rural African farming contexts.

## Methods

### Participants and procedure

Data for this school-randomized trial were collected across 100 schools and 200 classrooms in the Aboisso and Bouaflé regions of Cote d’Ivoire. A list of all of the schools in the two regions was obtained from the regional education offices. Fifty public schools within each region (*N* = 385 total in Aboisso, 612 in Bouafle) were selected by the district education office to participate in the study. Two waves of data were collected over one school year in September–October of 2018 (the start of the school year) and May–June 2019 (the end of the school year). All details of the experimental design and a pre-analysis plan were pre-registered at the AEA RCT Registry on October 31, 2018 (AEARCTR-0003385). The study was reviewed and approved by the Ethical Review Board at the University of Zurich (OEC IRB #2018-035).

One school could not be accessed for data collection due to remoteness. For the remaining 99 schools, class rosters of CP2 (equivalent to primary 2) and CE2 (equivalent to primary 4) were obtained. Twenty-five children were selected per school to participate in the assessments: 13 children were randomly chosen from the CP2 roster and 12 from the CE2 roster. Direct child assessments were conducted in school by trained enumerators. The parents of these children were also interviewed in person in their homes. In total, data was collected on 2475 children at baseline (1285 CP2 students and 1,190 CE2 students). In the spring, data was collected on 2243 (90.6%) of those children. The parents of each child were interviewed in person. Replacement households were selected in advance from class rosters in the event that parents could not be located for the interview. The final sample size was 2475 parents at baseline; an additional 25 parents were located and participated in the survey in the spring. All parents provided written informed consent for themselves and their child, and all children provided verbal assent, to participate in the study.

### The Eduq+ program

**Fig. 2:**
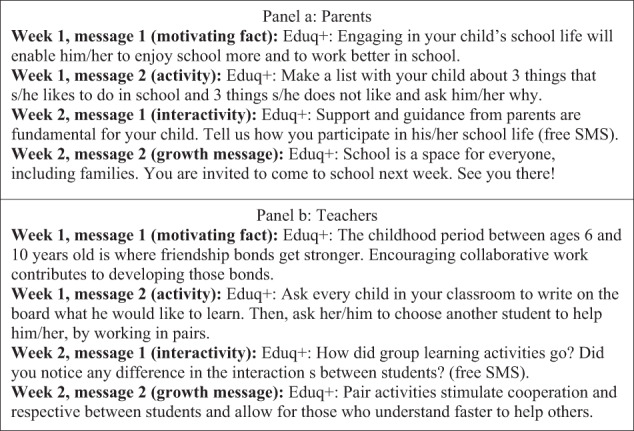
Sample sequence messages delivered to parents and teachers. Two sample weekly SMS sequences delivered to parents.

The Eduq+ program was implemented by Movva, a Brazilian social enterprise, in partnership with the Ministry of Education and participating schools. The adaptation process to the Ivorian context is detailed in the Supplementary Materials. There was an initial meeting at the school to introduce parents and teachers to the program. The program included two SMS-messages per week in simplified French, with behavioral “nudges” aimed at changing behavior. Messages were sent from December 2018 to June 2019. The messages include reminders, encouragement, and activities addressing information gaps, biased beliefs, and norms behind gender inequalities in education and broader development. Each core module is delivered over two weeks, with two messages per week, and structured as a specific sequence based on behavioral economic theory to induce behavior change—a motivating fact (message 1), a suggested activity (message 2), an interactive message (message 3), and a growth message (message 4). See Fig. 2 for an example sequence and Supplementary Materials for additional details.

For parents, messages related to supporting children’s social and emotional development and engagement in education at home and in school. No curricular knowledge was required. In this study, two platforms for message delivery were tested—text (in French) and audio (translated into the local language of the region). For teachers, only text messages in French were included. Message included content related to pedagogical tips tailored to the context (e.g., creating small group activities within large class sizes, discouraging corporal punishment, and encouraging them to attend to low-performing students), as well as motivating support to increase attendance and effort.

### Measures

#### Child outcomes

##### Literacy skills

Literacy in French was assessed using eight tasks measuring pre-literacy and literacy domains from two sources. Using the Early Grade Reading Assessment^46^, domains included letter-sound identification, nonword decoding, and word reading. Four additional adapted subtasks from EGRA were used and included phonological awareness, phoneme segmentation, synonyms, and antonyms^4^. Finally, one additional measure of phonological awareness from the International Development and Early Learning Assessment (IDELA)^47^ was included (*α* = 0.85).

##### Numeracy skills

Numeracy was assessed using eight subdomain tasks. Four tasks from the Early Grade Math Assessment^48^ included quantity discrimination, addition, subtraction, and missing number pattern identification. In addition, four tasks from the IDELA^47^ were used: number knowledge, one-to-one correspondence, shape identification, and sorting abilities based on color and shape (*α* = 0.86).

##### Child labor

Child labor activities were reported on both by children and parents. Children were asked four questions: “In the last month, have you engaged in one or more of the following activities for one hour or more?”^1^ Domestic work, such as buying food or cooking, cleaning the house, do the laundry, take care of children or other sick or old relatives^2^; Work in the fields, plot, or garden belonging to the family^3^; Engaged in any construction work or major repairs; and^4^ “Work in a cocoa plantation.” We rely on child-reported labor as research has shown this to be more accurate than parent reports^45^.

#### Mediators

##### Parental motivation

We adopted a measure of teacher autonomous motivation^49^ to measure parental motivation, which contained 9 items (*α* = 0.81). Because this measure had not been used in the rural Ivorian context, we conducted an exploratory factor analysis which yielded a clear 1-factor model with all items loading above 0.5. We extracted factor scores for each parent.

##### Parental engagement

Parental engagement in children’s social-emotional development and educational development was measured by nine items each, where parents reported how often they engaged in the following activities (1 = never, 2 = almost never, 3 = sometimes, 4 = almost always/always). Items for *Social-emotional Engagement* included play with (child), read a book to (child name), sing a song to (child), tell a story to (child) or ask (child) to tell a story, do any other recreational activity with (child), ask (child name) which activities and games he/she likes the most, ask (child) about his/her feelings, ask (child name) about his/her fears, and talk to (child) to help him/her managing her/his fears and difficulties (*α* = 0.72).

Items for *Educational Engagement* included: help (child) with homework or schoolwork, ask (child) if s/he did his/her homework or schoolwork, help (child) to organize school materials, incentivize (child) to not miss class or be late for school, ask (child) about his/her grades in tests, activities and classes, incentivize (child) to study or read, ask (child) about his/her day in school, go to school parent meetings, and talk to (child)’s teachers (*α* = 0.78).

#### Covariates

All covariates were measured at baseline. Demographics included grade-level fixed effects and child sex. For all models, identical assessments for all outcomes were collected at baseline and lagged baseline values of all outcomes and mediators were included in all regression models. Additional covariates at the child-level were also included.

##### School effort

Using 9 items from the Elementary School Motivation Scale^50^, school effort focused on children’s motivation for schooling and answered on a 5-point scale with 1 = always no and 5 = always yes”. According to the scale, the items were constructed in three sub-scale including intrinsic motivation (e.g., “I like going to school”), extrinsic motivation (e.g., “I go to school to please my parents or teacher”), and identified motivation (e.g., “Going to school allows me to learn many useful things”) (*α* = 0.67).

##### Social-emotional skills

Social-emotional skills were measured using IDELA social-emotional subscale^47^, with 14 items grouped into five constructs: self-awareness, emotion identification, perspective taking and empathy, friendship, and conflict/problem-solving. An example item of conflict solving involved asking children to imagine they are playing with a toy and that another child wants to play with the same toy, and then asking the children what they would do to resolve that conflict. “Correct” answers in the Ivorian context, as agreed upon by the assessors during training, included talking to the child, taking turns, sharing, and getting another toy (*α* = 0.62).

##### Executive function

*Cognitive flexibility* was assessed using a tablet-based Hearts and Flowers task^51^ (*α* = 0.86). *Short-term memory* was measured using a visual digit span, where children were shown seven series of numbers ranging from two to seven digits and asked to write down the numbers they saw in the same order after each series was presented (adapted from ref. ^52^; *α* = 0.79).

##### Impulsive behavior

Impulsivity was measured using an 8-item scale, where children reported how often they had engaged in a set of behaviors during the past school year (1 = almost never, 2 = about once/month, 3 = account 3 times/month, 4 = about once a week, and 5 = at least once a day)^17^. Example items included “I interrupted other students while they were talking”, “I lost my temper at home or at school”, and “I talked back to my teacher or parent when I was upset” (*α* = 0.83).

##### Self-esteem

We used two items from the Harter Self-perception Profile of Children^53^. Children were asked: “When you think about yourself, how do you feel” and “How do you feel about the way you are?” Response options were 1 = really sad, 2 = sad, 3 = neutral, 4 = happy, 5 = really happy (*r* = 0.52).

##### Fixed mindset

Fixed mindset was measured using three items from the Measures of Motivational Frameworks^54^ using a response scale of 1–5, where 1 = strongly disagree and 5=strongly agree. Question included: “Imagine a child who thinks that a person is a certain amount smart and can’t change very much. How much do you agree with this child?”; “Imagine a child who thinks that being good or not at math is something that you really can’t change. Some people are good at math and other people aren’t,” and “Do you think that if someone has to try really hard in a subject in school, it means s/he can’t be good at that subject?”. Higher values indicated a higher fixed mindset (*α* = 0.64).

### Analytic strategy

#### Randomization

In the summer of 2018, schools were randomly assigned, stratified by region, to (i) parent text messages only (*N* = 13 schools, 26 classrooms), (ii) parent audio message only (*N* = 13, 26 classrooms), (iii) parent text messages plus teacher text messages (*N* = 12 schools, 24 classrooms), (iv) parent audio messages plus teacher text messages (*N* = 12 schools, 24 classrooms), and (v) a control group (*N* = 50 schools, 100 classrooms). There were no differential effects of delivery mode, and for this analysis, we pool both modes (*N* = 26 schools/52 classrooms for parent messages alone, *N* = 24 schools/48 classrooms for parent and teacher messages combined).

#### Baseline equivalency

We conducted a baseline equivalency analysis to ensure that the randomization yielded statistically equivalent treatment and control groups based on observable characteristics. We tested whether the mean values for a set of school, teacher, and child characteristics differed by treatment group. We found a balance on baseline characteristics across treatment groups (see Table 6).

**Table 6:** Baseline equivalency across treatment groups.

	Sub-sample means				ANOVA test *p*-value	# of obs.
	Control	Parents alone	Teachers alone	Both		
*Panel A: School chars*						
Number of students enrolled	258.44	281.83	283.48	273.35	0.714	99
Number of teachers	6.20	5.57	5.72	6.19	0.073	99
Hot days	13.48	15.43	14.36	10.62	0.158	99
Meals provided	0.56	0.52	0.60	0.31	0.187	99
Chalks	2.52	2.52	2.76	2.58	0.743	99
Blackboards	2.16	2.26	2.16	2.00	0.756	99
Textbooks	3.48	3.35	3.52	3.50	0.798	99
Notebooks	2.20	2.52	2.60	2.77	0.180	99
Pens	2.44	2.52	2.84	2.88	0.354	99
Monthly teacher absenteeism	1.64	1.35	1.32	1.35	0.524	99
Daily students’ absence	7.12	11.22	11.16	8.38	0.653	99
*F*-test for nudges to parents					0.524	99
*F*-test for nudges to teachers					0.793	99
*F*-test for nudges to both					0.226	99
*Panel B: Caregiver chars*						
Female share	0.42	0.37	0.45	0.40	0.190	2471
Relationship with child	3.08	3.30	3.03	3.24	0.323	2471
Marital status	1.98	1.98	2.05	2.02	0.254	2471
Years as primary caregiver	5.89	6.20	6.52	5.94	0.110	2441
Source of income	3.65	3.76	3.79	3.89	0.901	2,71
Ever attended school	1.38	1.41	1.42	1.43	0.575	2471
Std. parental engagement	−0.01	−0.04	−0.05	−0.07	0.628	2471
Std. mindset wrt children	−0.07	−0.05	0.00	−0.01	0.627	2471
Std. failure mindset	−0.02	−0.07	−0.09	−0.06	0.578	2471
Std. parental aspirations	−0.01	0.02	−0.11	−0.03	0.314	2471
Std. parental expectations	−0.04	−0.01	−0.07	−0.03	0.794	2471
Beliefs child attendance	0.61	0.61	0.57	0.65	0.887	2454
Std. parents’ mental health	0.00	−0.08	−0.06	−0.08	0.106	2471
Std. physical punishment	0.04	0.09	0.11	0.01	0.104	2471
Std. child labor	0.01	0.07	0.02	0.09	0.503	2,471
Std. audio trust	0.00	0.02	0.03	0.07	0.833	2408
Std. audio comprehension	0.00	−0.06	0.00	0.02	0.599	2408
Std. visual trust	0.00	−0.07	−0.05	0.04	0.427	2408
Std. visual comprehension	0.00	−0.02	0.04	0.07	0.601	2408
Std. audio memory	0.00	−0.04	−0.02	−0.02	0.675	2475
Std. visual memory	0.00	−0.03	−0.12	−0.01	0.810	2475
Std. audio attention	0.00	0.05	0.03	0.03	0.532	2475
Std. visual attention	0.00	0.04	0.01	−0.03	0.224	2475
*F*-test for nudges to parents					0.512	2362
*F*-test for nudges to teachers					0.291	2362
*F*-test for nudges to both					0.582	2362
*Panel C: Child characteristics*						
Std. literacy	−0.13	−0.12	−0.11	−0.04	0.369	2475
Std. numeracy	−0.14	−0.12	−0.10	−0.08	0.770	2475
Std. social-emotional	−0.03	0.01	−0.03	−0.03	0.869	2475
Std. working memory	−0.11	−0.10	−0.09	−0.09	0.936	2475
Female share	0.47	0.54	0.50	0.50	0.123	2475
Std. child labor	−0.01	−0.05	−0.03	−0.07	0.689	2475
Std. self-esteem	0.04	0.01	0.05	0.02	0.869	2475
Std. mindset	−0.06	−0.11	−0.03	−0.08	0.649	2475
Teacher absenteeism	0.11	0.11	0.12	0.11	0.936	2104
Std. parental engagement	−0.08	−0.13	−0.03	−0.04	0.162	2475
Std. student effort	−0.09	−0.06	−0.06	−0.03	0.539	2475
*F*-test for nudges to parents					0.115	2104
*F*-test for nudges to teachers					0.797	2104
*F*-test for nudges to both					0.542	2104
*Panel D: Teacher characteristics*						
Age	41.29	38.70	38.28	37.71	0.198	197
Years worked as teacher	14.88	11.48	10.74	10.25	0.097	197
Years worked at this school	7.04	5.22	5.06	5.52	0.423	197
Education	3.88	4.26	4.18	4.23	0.168	197
Completed the last level	1.53	1.43	1.42	1.37	0.408	197
Std. language skills	0.00	−0.34	−0.39	−0.19	0.065	197
Follow advice from SMS	3.92	3.67	3.80	3.83	0.134	197
Std. external motivation	0.02	0.24	0.01	0.00	0.280	197
Std. introjected motivation	−0.02	0.05	−0.19	−0.12	0.374	197
Std. intrinsic motivation	0.04	0.10	−0.04	−0.03	0.756	197
Std. identified motivation	−0.05	0.05	−0.13	−0.14	0.592	197
Std. failure mindset	−0.05	−0.14	−0.19	−0.01	0.445	197
Std. job satisfaction	0.10	0.09	−0.04	−0.05	0.635	197
Teacher self-reported attendance	2.35	2.33	2.50	1.98	0.776	196
Arrive late	1.06	0.80	0.72	0.79	0.890	197
Leave early	0.14	0.11	0.16	0.27	0.674	197
*F*-test for nudges to parents					0.466	196
*F*-test for nudges to teachers					0.870	196
*F*-test for nudges to both					0.743	196

#### Sample attrition and weighting

Of the original 2475 students at baseline, 2246 (90.6%) were assessed at follow-up. We use attrition weights to handle selective attrition. First, we predicted the probability of successfully tracking control group respondents at the end-line survey using a logistic regression based on baseline characteristics. Baseline characteristics included in this model were child sex; standardized baseline grades (numeracy and literacy); standardized parental engagement; standardized student effort; standardized child labor; standardized socio-emotional skills; standardized working memory; standardized visual attention; standardized impulsivity; standardized self-esteem; standardized mindset. These estimates were then used to predict propensity scores for each observation in the whole sample (treatment and control). We then use inverse propensity weights in all regression models. This matching procedure ensures that our estimates capture causal treatment effects (rather than compositional changes across treatment groups) under the assumption that selection is independent of potential outcomes once conditioning on observable baseline characteristics.

#### Impact estimates

##### Power calculations

Given the sample design, with 80% power and 10% statistical significance, post hoc power estimates point to a minimum detectable effect size of 0.18 on the learning summary measure on 0.22 on child labor.

##### Impact analysis

For the impact analysis, we create standardized scale scores for each outcome^55^. First, child outcomes, we standardized each component within grade (normalizing by the mean and standard deviation of the control group) and then created an average score across the components to construct a summary variable. For parent outcomes, we standardize each composite score by the mean and standard deviation of the control group.

We then used a between-group analysis of covariance (ANCOVA) regression to examine treatment group impacts, including the full set of covariates in each model, with a treatment status categorical variable (0 = control, 1 = parents only, 2 = teachers only, 3 = parents and teachers). Standard errors were clustered at the school-level with fixed effects for grades. We present the results for the full sample of children combined, and exploratory follow-up analyses by subgroups adding an interaction term between treatment status and each moderator.

### Reporting summary

Further information on research design is available in the Nature Research Reporting Summary linked to this article.

### Supplementary information

Supplementary Materials

Reporting Summary

## Data Availability

The data that support the findings of this study are publicly available and can be downloaded at the following location: https://osf.io/hx8ve/.

## Code availability

The Stata code used to conduct all analyses for this study is available upon request from the corresponding author, S.W.
