# Supplementary material for: Nudging parents and teachers to improve learning and reduce child labor in Cote d’Ivoire
Source: NPJ Sci Learn. 2023 Sep 13;8:37. doi: 10.1038/s41539-023-00180-z (PMC10499780; doi:10.1038/s41539-023-00180-z)
Supplement: Supplementary file 1 — Supplementary Materials [file 41539_2023_180_MOESM1_ESM.pdf]

Supplementary Notes

Literature Review on Child Labor and Education

Both theoretically and empirically, it is not clear whether child labor substantially displaces schooling. In rural settings, many children balance both labor on family farms and school (Sadhu et al., 2020). Further, in both urban and rural parts of sub-Saharan Africa, domestic work is extremely common among children (e.g., Haile & Haile, 2012; Kembou et al., 2022). Child labor can be an obstacle to school attendance and learning. Work responsibilities related to farming specifically may lead to forced school dropout temporarily during harvest season (Tulane University, 2015). In other cases, work may result in fatigue or other negative health impacts, or just leave little time for school-related work.

Correlational evidence indicates that for children who combine work and schooling, educational attainment is likely to be lower. For example, factors that predicted an increase in child labor also predicted a decrease in school attendance and increase in grade repetition in Paraguay (Patrinos & Psacharopoulos, 1995), and two studies found that child work directly predicted lower reading and mathematics attainment in Tanzania and Ghana (Akabayashi & Psacharopoulos, 2007; Heady, 2003). Specifically, Akabayashi and Psacharopoulos (1999) showed that school attendance and children's reading skill decreases with increased child labor hours. Further, across nine countries in Latin America, (Gunnarsson et al. (2006) found that child labor lowers test both literacy and numeracy test scores. Working one standard deviation above the mean was associated with average scores that were 16% lower in mathematics and 11% lower in language. Importantly, most of this evidence is from before the Millennium Development Goals in 2000, after which a large increase in primary school enrollment occurred throughout sub-Saharan Africa and globally. In more recent data from 2021, Kembou and colleagues (2022) found that the majority of Ivorian children in their sample balanced both work and school, and a very small portion of the sample worked without simultaneously

attending school.

There have been a few quasi-experimental studies in the past two decades examining the causal connection between child labor and school outcomes. Boozer and Suri (2001) exploited regional variation in rainfall in Ghana and found that a one-hour increase in child labor led to a decrease in contemporaneous schooling of about 0.4 hours. Beegle et al. (2004) instrumented for participation in child labor in Vietnam with rice prices, a variable that influences child labor but is plausibly exogenous with respect to household choices. They found that the mean level of child labor leads to a 46 percent reduction in the probability of being in school, a 21 percent decrease in educational attainment, and a doubling in the probability of working for wages five years later. In Bangladesh, Ravallion and Wodon (2000) examined random variation in participation in a food-for-school program, finding that the program led to a significant increase in school participation. Yet when probing further, they found that only a small fraction of the increased hours in school were attributed to a decrease in child labor, suggesting that child labor and schooling were not fully linked.

A much less explored aspect is the role of teachers in child labor. If parents perceive the returns to education as low, they may be more likely to engage their children in labor activities at the expense of school. Poor teacher attendance may signal low educational quality to parents and could also lead children to drop out of school if they attend but do not learn much. A recent study covering 258 cocoa-growing communities in Ghana and Cote d'Ivoire examined the link between school access, infrastructure, services, and school management. Using an educational quality index across several dimensions of the school context, the study found a link between educational quality and child labor. Specifically, in communities with the highest quality of education score, child labor prevalence was 66% lower than in the communities with the lowest quality of education score(International Cocoa Initiative, 2019).

Supplementary Methods

Community Engagement and Program Adaptation

The EDU+ program was originally developed for parents in Brazil. Overall, it aims to tackle cognitive and behavioral barriers to parent engagement by increasing parental salience and knowledge around the importance of their engagement in child education through information and practical suggestions of activities to incorporate in their day-to-day routines. Messages included, among other topics, setting time for homework, talking with children about their future educational plans, and encouraging parents to ask children about their experiences in school. No curricular knowledge was required for parents to follow the suggestions included in the messages. Messages also targeted the development of social-emotional skills, with themes such as positive discipline, growth mindset, and fostering communication at home. The program was designed on the model of Ready4K! (Cortes et al. 2021; York, Loeb, and Doss 2019) and co-developed by Movva Technologies (an ed-tech start-up).

The program in Cote d'Ivoire was adapted to address the specific challenges to parent-child relationships and school engagement in the Ivorian focal communities and to the West African context more generally. Two organizations with substantial experience supporting families on child development in Africa were consulted for recommendations on how to approach the child development activities with parents based on their proven experiences. These included the International Rescue Committee (working through West Africa) and Lively Minds (working in rural communities in Ghana). The main recommendations were: i) drawing on the scientific basis for child development (and brain development, specifically) is more convincing to parents; and ii) encourage parents to speak with and learn from other parents. Messages were adapted to incorporate scientific evidence as part of the motivating facts at the start of sequences, and messages were rewritten in the local French dialect and local languages (for audio messages).

The level of language complexity targeted was that of a public-school parent, and the first batch of ten messages were written in the voice of a parent sharing his experiences since he learned about the scientific evidence on children's development.

Engagement and consultation started in November 2018, and in parallel additional organizations were consulted, including Graines de Paix and the Global Initiative to end all corporal punishment of Children (GIEACPC), which supported us in the production of new content (5 sequences) focusing on increasing awareness about the negative impacts of corporal punishment, bringing the perspective of positive discipline.

The engagement messages were translated to a simple version of French ("French Facile") and to other 6 local languages – Agni, Baoulé, Gouro, Malinke, Moré, Yahouré – and piloted with a group of parents from the local communities and deemed acceptable. Audio messages were then recorded in all of the local languages.

Supplementary References

Akabayashi, H., & Psacharopoulos, G. (2007). The trade-off between child labour and human capital formation: A Tanzanian case study. *The Journal of Development Studies*, *35*(5), 120–140. https://doi.org/10.1080/00220389908422594

Beegle, K., Dehejia, R., Gatti, R., & Dehejia, R. (2004). *Why Should We Care About Child Labor? The Education, Labor Market, and Health Consequences of Child Labor*. https://doi.org/10.3386/W10980

Boozer, M., & Suri, T. (2001). Child Labor and Schooling Decisions in Ghana. *Yale University Working Paper*.

Cortes, K., Hans Fricke, E., Loeb, S., Song, D.S., & York, B.N. (2021). Too Little or Too Much? Actionable Advice in an Early-Childhood Text Messaging Experiment. *Education Finance and Policy,* 16(2), 209–32. doi: 10.1162/edfp_a_00304.

Gunnarsson, V., Orazem, P. F., & Sánchez, M. A. (2006). Child Labor and School Achievement in Latin America. *World Bank Economic Review*, *20*(1), 31–54. https://doi.org/10.1093/WBER/LHJ003

Haile, G., & Haile, B. (2012) Child labour and child schooling in rural Ethiopia: nature and trade-off. *Education Economics, 20*(4), 365-385, DOI: 10.1080/09645292.2011.623376

Heady, C. (2003). The Effect of Child Labor on Learning Achievement. *World Development*, *31*(2), 385–398. https://doi.org/10.1016/S0305-750X(02)00186-9

International Cocoa Initiative. (2019). *Education quality and child labour: A review of evidence from cocoa-growing communities in Côte d'Ivoire and Ghana*. https://www.cocoainitiative.org/knowledge-hub/resources/education-quality-and-child-labour-review-evidence-cocoa-growing

Kembou, S. N., Wolf, S., Jasinska, K., & Ogan, A. (2022). *Child labor and schooling in rural Côte d'Ivoire: Incidence and determinants*.

Patrinos, H. A., & Psacharopoulos, G. (1995). Educational Performance and Child Labor in Paraguay. *International Journal of Educational Development*, *15*(1).

Ravallion, M., & Wodon, Q. (2000). Does Child Labour Displace Schooling? Evidence on Behavioural Responses to an Enrollment Subsidy. *The Economic Journal*, *110*(462), 158–175. https://doi.org/10.1111/1468-0297.00527

Sadhu, S., Kysia, K., Onyango, L., Zinnes, C., Lord, S., Monnard, A., & Arellano, I. R. (2020). *Assessing Progress in Reducing Child Labor in Cocoa Production in Cocoa Growing Areas of Côte d'Ivoire and Ghana NORC*. Chicago, IL.

Tulane University. (2015). *FINAL REPORT 2013/14 Survey Research on Child Labor in West African Cocoa Growing Areas*.

York, B., Loeb, S., & Doss, C. (2019). One Step at a Time: The Effects of an Early Literacy Text-Messaging Program for Parents of Preschoolers. *Journal of Human Resources*. doi: 10.3368/jhr.54.3.0517-8756R.

Supplementary Table 1

Impacts on Child Labor Disaggregated by Activity

	Domestic labor	Family fields	Constructio n work	Cocoa fields
Parents	0.0465*	0.0171	0.0525**	0.0514
	(0.0242)	(0.0385)	(0.0242)	(0.0500)
Teachers	-0.0057	0.0064	0.0016	0.0236
	(0.0239)	(0.0417)	(0.0241)	(0.0519)
Both	0.0402	-0.0535	-0.0242	-0.0024
	(0.0190)	(0.0467)	(0.0215)	(0.0531)
Observations	2246	2246	2246	2246
R-squared	0.0341	0.1454	0.0338	0.1196
Control mean endline	0.878	0.693	0.087	0.498
Parents = Both [p-value]	0.755	0.188	0.002	0.376
Teachers = Both [p-value]	0.027	0.288	0.276	0.677

Notes: Parents = 1 in schools where parents alone receive message; Teachers = 1 in schools where teachers alone receive messages; Both = 1 in schools where both parents and teachers receive messages. Estimates are derived using between group analysis of covariance (ANCOVA) regression to examine treatment group impacts, including the full set of covariates in each model, with a treatment status categorical variable. In all columns, observations are weighted by the inverse of the predicted probability of being tracked at endline, computed using baseline students' characteristics in the control group. Baseline controls include grade level; child sex; standardized baseline grades (numeracy and literacy); standardized parental engagement; standardized student effort; standardized child labor composite; standardized socio-emotional skills; standardized working memory; standardized visual attention; standardized impulsivity; standardized self-esteem; standardized mindset. Standard errors clustered at the school level in parentheses. * $p < .10$; ** $p < .05$
