# Supplementary material for: Nudging parents and teachers to improve learning and reduce child labor in Cote d’Ivoire
Source: NPJ Sci Learn. 2023 Sep 13;8:37. doi: 10.1038/s41539-023-00180-z (PMC10499780; doi:10.1038/s41539-023-00180-z)
Supplement: Supplementary file 2 — Reporting Summary [file 41539_2023_180_MOESM2_ESM.pdf]

nature portfolio

Corresponding author(s): Sharon Wolf

Last updated by author(s): Jul 26, 2023

Reporting Summary

Nature Portfolio wishes to improve the reproducibility of the work that we publish. This form provides structure for consistency and transparency in reporting. For further information on Nature Portfolio policies, see our Editorial Policies and the Editorial Policy Checklist.

Statistics

For all statistical analyses, confirm that the following items are present in the figure legend, table legend, main text, or Methods section.

n/a	Confirmed	
☐	☒	The exact sample size (n) for each experimental group/condition, given as a discrete number and unit of measurement
☐	☒	A statement on whether measurements were taken from distinct samples or whether the same sample was measured repeatedly
☐	☒	The statistical test(s) used AND whether they are one- or two-sided<br>*Only common tests should be described solely by name; describe more complex techniques in the Methods section.*
☐	☒	A description of all covariates tested
☐	☒	A description of any assumptions or corrections, such as tests of normality and adjustment for multiple comparisons
☐	☒	A full description of the statistical parameters including central tendency (e.g. means) or other basic estimates (e.g. regression coefficient) AND variation (e.g. standard deviation) or associated estimates of uncertainty (e.g. confidence intervals)
☐	☒	For null hypothesis testing, the test statistic (e.g. F, t, r) with confidence intervals, effect sizes, degrees of freedom and P value noted<br>*Give P values as exact values whenever suitable.*
☒	☐	For Bayesian analysis, information on the choice of priors and Markov chain Monte Carlo settings
☐	☒	For hierarchical and complex designs, identification of the appropriate level for tests and full reporting of outcomes
☐	☒	Estimates of effect sizes (e.g. Cohen's d, Pearson's r), indicating how they were calculated

Our web collection on statistics for biologists contains articles on many of the points above.

Software and code

Policy information about availability of computer code

Data collection	SurveyCTO was used to collect all data for this study.
Data analysis	All analyses were conducted in Stata v18.

For manuscripts utilizing custom algorithms or software that are central to the research but not yet described in published literature, software must be made available to editors and reviewers. We strongly encourage code deposition in a community repository (e.g. GitHub). See the Nature Portfolio guidelines for submitting code & software for further information.

Data

Policy information about availability of data

All manuscripts must include a data availability statement. This statement should provide the following information, where applicable:

- Accession codes, unique identifiers, or web links for publicly available datasets
- A description of any restrictions on data availability
- For clinical datasets or third party data, please ensure that the statement adheres to our policy

The data that support the findings of this study are publicly available and can be downloaded at the following location: https://osf.io/hx8ve/

Research involving human participants, their data, or biological material

Policy information about studies with human participants or human data. See also policy information about sex, gender (identity/presentation), and sexual orientation and race, ethnicity and racism.

Reporting on sex and gender	We use the term sex to refer to biological attributes of males and females. We use the term gender when discussing bias and discrimination (e.g., gender bias).
Reporting on race, ethnicity, or other socially relevant groupings	N/A
Population characteristics	Children were 47% female and evenly distributed between primary school grades 2 and 4. Parents/primary caregivers were 42% female, 98% married, and 62% ever attended any level of school.
Recruitment	Data for this school-randomized trial were collected across 100 schools and 200 classrooms in the Aboisso and Bouaflé regions of Cote d'Ivoire. A list of all of schools in the two regions was obtained from the regional education offices. Fifty public schools within each region (N = 385 total in Aboisso, 612 in Bouafle) were selected by the district education office to participate in the study. Two waves of data were collected over one school year in September-October of 2018 (start of the school year) and May-June 2019 (end of the school year). All details of the experimental design and a pre-analysis plan were pre-registered at the AEA RCT Registry on October 31, 2018 (AEARCTR-0003385). The study was reviewed and approved by the Ethical Review Board at the University of Zurich. One school could not be accessed for data collection due to remoteness. For the remaining 99 schools, class rosters of CP2 (equivalent to primary 2) and CE2 (equivalent to primary 4) were obtained. Twenty-five children were selected per school to participate in the assessments: 13 children were randomly chosen from the CP2 roster and 12 from the CE2 roster. Direct child assessments were conducted in school by trained enumerators. The parents of these children were also interviewed in person in their homes. In total, data was collected on 2,475 children at baseline (1,285 CP2 students and 1,190 CE2 students). In the spring, data was collected on 2,243 (90.6%) of those children. The parents of each child were interviewed in person. Replacement households were selected in advance from class rosters in the event that parents could not be located for the interview. The final sample size was 2,475 parents at baseline; an additional 25 parents were located and participated in the survey in the spring. All parents provided written informed consent for themselves and their child, and all children provided verbal assent, to participate in the study.
Ethics oversight	University of Zurich

Note that full information on the approval of the study protocol must also be provided in the manuscript.

Field-specific reporting

Please select the one below that is the best fit for your research. If you are not sure, read the appropriate sections before making your selection.

☐ Life sciences ☒ Behavioural & social sciences ☐ Ecological, evolutionary & environmental sciences

For a reference copy of the document with all sections, see nature.com/documents/nr-reporting-summary-flat.pdf

Behavioural & social sciences study design

All studies must disclose on these points even when the disclosure is negative.

Study description	School-randomized trial using quantitative data analysis.
Research sample	Children and their parents from 100 schools in the Aboisso and Bouaffle regions of Cote d'Ivoire.
Sampling strategy	A list of all of schools in the two regions was obtained from the regional education offices. Fifty public schools within each region were selected by the district education office to participate in the study. One school could not be accessed for data collection due to remoteness. For the remaining 99 schools, class rosters of CP2 (equivalent to primary 2) and CE2 (equivalent to primary 4) were obtained. Twenty-five children were selected per school to participate in the assessments: 13 children were randomly chosen from the CP2 roster and 12 from the CE2 roster.
Data collection	Two waves of data were collected over one school year in September-October of 2018 (start of the school year) and May-June 2019 (end of the school year). Direct child assessments were conducted in school by trained enumerators. The parents of these children were also interviewed in person in their homes. In total, data was collected on 2,475 children at baseline (1,285 CP2 students and 1,190 CE2 students). In the spring, data was collected on 2,243 (90.6%) of those children. The parents of each child were interviewed in person. Replacement households were selected in advance from class rosters in the event that parents could not be located for the interview. The final sample size was 2,475 parents at baseline; an additional 25 parents were located and participated in the survey in the spring. All parents provided written informed consent for themselves and their child, and all children provided verbal assent, to participate in the study.

Timing	September-October of 2018 (start of the school year) and May-June 2019 (end of the school year).
Data exclusions	One school was excluded due to its remoteness and inability to be accessed for data collection.
Non-participation	Of the original 2,475 students at baseline, 2,246 (90.6%) were assessed at follow-up.
Randomization	School-randomization

Reporting for specific materials, systems and methods

We require information from authors about some types of materials, experimental systems and methods used in many studies. Here, indicate whether each material, system or method listed is relevant to your study. If you are not sure if a list item applies to your research, read the appropriate section before selecting a response.

Materials & experimental systems

n/a	Involved in the study
☒	☐ Antibodies
☒	☐ Eukaryotic cell lines
☒	☐ Palaeontology and archaeology
☒	☐ Animals and other organisms
☒	☐ Clinical data
☒	☐ Dual use research of concern
☒	☐ Plants

Methods

n/a	Involved in the study
☒	☐ ChIP-seq
☒	☐ Flow cytometry
☒	☐ MRI-based neuroimaging
